# High dietary zinc feeding promotes persistence of multi-resistant *E*. *coli* in the swine gut

**DOI:** 10.1371/journal.pone.0191660

**Published:** 2018-01-26

**Authors:** Lisa Ciesinski, Sebastian Guenther, Robert Pieper, Martin Kalisch, Carmen Bednorz, Lothar H. Wieler

**Affiliations:** 1 Centre for Infection Medicine, Institute of Microbiology and Epizootics, Department of Veterinary Medicine, Freie Universität Berlin, Berlin, Germany; 2 Institute of Animal Nutrition, Department of Veterinary Medicine, Freie Universität Berlin, Berlin, Germany; 3 Robert Koch Institute, Berlin, Germany; Humboldt-Universitat zu Berlin, GERMANY

## Abstract

High levels of zinc oxide are used frequently as feed additive in pigs to improve gut health and growth performance and are still suggested as an alternative to antimicrobial growth promoters. However, we have recently described an increase of multi-resistant *E*. *coli* in association to zinc feeding in piglets. This previous study focused on clonal diversity of *E*. *coli*, observing the effect on multi-resistant strains by chance. To shed further light into this highly important topic and falsify our previous findings, we performed a zinc pig feeding trial where we specifically focused on in-depth analysis of antimicrobial resistant *E*. *coli*. Under controlled experimental conditions, piglets were randomly allocated to a high dietary zinc (zinc group) and a background zinc feeding group (control group). At different ages samples were taken from feces, digesta, and mucosa and absolute *E*. *coli* numbers were determined. A total of 2665 *E*. *coli* isolates were than phenotypically tested for antimicrobial resistance and results were confirmed by minimum inhibitory concentration testing for random samples. In piglets fed with high dietary zinc, we detected a substantial increase of multi-resistant *E*. *coli* in all gut habitats tested, ranging from 28.9–30.2% multi-resistant *E*. *coli* compared to 5.8–14.0% in the control group. This increase was independent of the total number of *E*. *coli*. Interestingly, the total amount of the *E*. *coli* population decreased over time. Thus, the increase of the multi-resistant *E*. *coli* populations seems to be linked with persistence of the resistant population, caused by the influence of high dietary zinc feeding. In conclusion, these findings corroborate our previous report linking high dietary zinc feeding of piglets with the occurrence of antimicrobial resistant *E*. *coli* and therefore question the feeding of high dietary zinc oxide as alternative to antimicrobial growth promoters.

## Introduction

The European ban of antibiotics as feed additives in 2006 (REGULATION (EC) No 1831/2003) has enforced the search for possible alternatives to prevent frequently occurring post-weaning diarrhea in pigs. Besides possible alternatives such as probiotics, prebiotics, enzymes, herbal products, organic acids, and other bioactive compounds, cationic trace elements such as zinc from zinc oxide at pharmacological levels have been propagated frequently by science and industry [1–3].

Although resistance against antimicrobial substances is a naturally occurring phenomenon in commensal and pathogenic bacteria [4, 5], the immense use and misuse of antimicrobial substances in human- and veterinary medicine led (i) to the selection for antimicrobial resistant bacteria and (ii) to the acquisition and spread of resistance genes to pathogens [6, 7]. However, to maintain a therapeutic efficiency of current antimicrobials, it is of utmost importance to reduce the spread of antimicrobial resistance genes. The ban of antimicrobial growth promoters clearly helped to minimize the use of antimicrobials [8], but the resulting massive use of zinc oxide for maintaining gut health and support growth promotion might have consequences which so far have only been poorly investigated.

The trace element zinc is an essential ingredient of food and feed. It is a cofactor for more than 300 enzymes [9, 10] and crucial for cell growth, DNA synthesis, cell division, immune system function, cognitive functions, and blood clotting [11]. The current dietary recommendations for growing pigs range between 80 to 100 mg zinc/kg diet and within the EU the current maximum allowance level is 150 mg zinc/kg diet (REGULATION (EC) No 1334/2003). However, as veterinary medicinal product for the post-weaning period, different formulations with ≥2500 mg zinc oxide/kg diet are approved for a period of 14 days [12] and outside the EU high doses of zinc oxide with concentrations from 2000–3000 mg zinc/kg diet are widely used as in-feed additive with even less regulations to control diarrhea in young pigs [13–15]. Positive effects through high dietary zinc feeding dominate during the first 10 to 14 days post-weaning [16, 17], but positive effects have also been detected for a feeding period of 28 days [18, 19]. This knowledge potentially encourages feeding durations longer than 14 days in less regulated countries or under conditions of recurrent periods of diarrhea and low performance.

In a previous zinc feeding trial with piglets fed high levels of dietary zinc oxide, we observed an increase of multi-resistant intestinal bacteria. In detail, we found that 18.6% of the *Escherichia* (*E*.) *coli* clones isolated from a high zinc group (2425 mg zinc/kg diet) were multi-resistant but not a single clone from the control group (57 mg zinc/kg diet) [20]. In addition, an increase of the copy numbers for tetracycline and sulfonamide resistance genes was recently observed in the gut of piglets fed high dietary zinc oxide after the weaning [21].

Thus, high dietary zinc feeding may increase the occurrence of antimicrobial resistance, as zinc-tolerance is linked to antimicrobial resistance [22], a finding that is gaining increasing recognition [23–25]. In livestock, recent publications connect the zinc concentration in liquid pig manure with phenotypic antimicrobial resistance in *E*. *coli* [26] and feeding of pharmacological zinc levels was associated with an increased prevalence and persistence of methicillin-resistant *Staphylococcus aureus* [27]. Thus, apart from the environmental pollution by high concentrations of zinc its use as an alternative for antimicrobial growth promoters is highly questionable.

Multi-resistant bacteria are a problem of worldwide health relevance with limited treatment options leading to longer treatment duration, an increased morbidity and mortality and thus to increasing treatment costs [7, 28]. Giving regard to the One health concept [29] it is necessary to investigate the influence of high dietary zinc feeding on the resistance status of gut bacteria from livestock.

To corroborate the effect of zinc on multi-resistance *in vivo* and to substantiate own recent results we intended to deepen our understanding on the population dynamics of *E*. *coli* under the influence of high dietary zinc feeding. Therefore, by applying a modified study design we comparatively fed piglets with high (zinc group) and background zinc concentrations (control group) concentrating on: (i) investigation of phenotypic antimicrobial resistance using antimicrobial selective plates without a prior clonal selection to focus on antimicrobial resistance (ii) parallel analysis of the resistant and overall numbers of *E*. *coli* (iii) expansion of the sampling scheme to feces, digesta, and mucosa samples as well as (iv) determination of the status quo regarding resistance of the *E*. *coli* population before weaning and the start of the zinc feeding.

Our data revealed an increasing effect of high dietary zinc feeding on the percentage of multi-resistant *E*. *coli* isolates and therefore substantiate recent findings.

## Materials and methods

### Animals, housing, and diets

Offspring of animals and experimental conditions have been described in previous studies [20, 21, 30]. Briefly, a total of 32 landrace piglets were weaned 25±1 days after birth and randomly allocated to two different feeding groups balancing for gender, litter, and body weight. Animals were housed in flatdeck pens with ad libitum access to feed and water. Diets were based on wheat, barley, corn, and soybean meal and were described previously [30]. Corn starch was partially replaced by analytical grade zinc oxide (Sigma Aldrich, Taufkirchen, Germany), which was determined by atomic absorption spectrometry (AAS vario 6 spectrometer; Analytik Jena, Jena, Germany) resulting in a final concentration of 72 mg zinc/kg diet (control) and 2103 mg/kg (zinc group) respectively. All feed was produced at the experimental site under controlled conditions. As pure corn starch with low inclusion/replacement level was replaced by zinc oxide for zinc content adjustment we suspect that the differences in biological zinc in the corn are so low to be neglectable. No antimicrobial substances were administered to the piglets or their mothers. The experimental trial followed the institutional and national guidelines for the care and use of animals and was approved by the local state office of occupational health and technical safety ‘Landesamt für Gesundheit und Soziales, Berlin’ (LaGeSo Reg. Nr. 0296/13).

### Sampling

**Fig 1:**
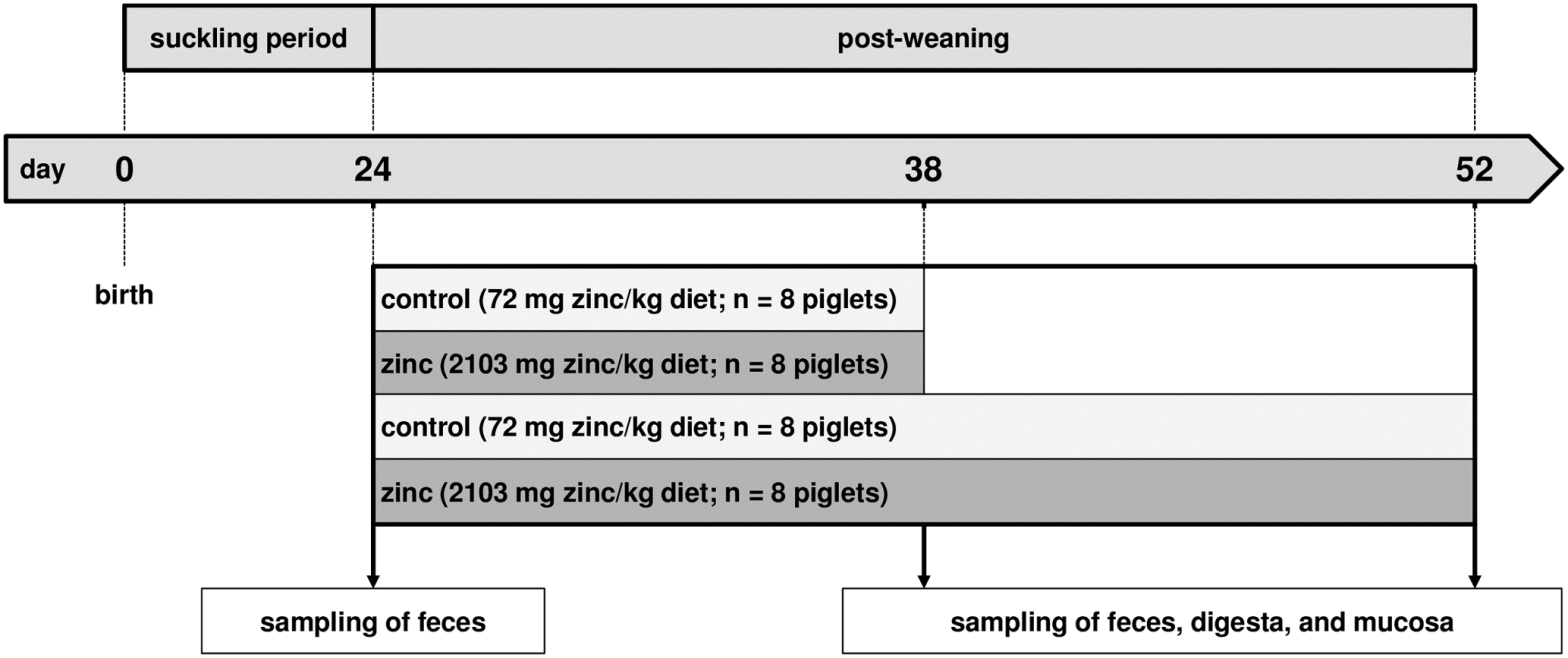
Schematic diagram of the zinc feeding trial. Piglets were weaned 25±1 days after birth. N values present the number of animals per feeding group and experimental stage. Arrows indicate the sampling time points linked to the pig age and habitat of taken samples.

This study investigated the occurrence of *E*. *coli* in three different habitats: feces as well as digesta and mucosa samples from the colon ascendens as main *E*. *coli* habitat at three different time points (Fig 1). At the age of 24 days (one day before weaning) feces were collected of all 32 piglets to investigate the status quo of the feces *E*. *coli* population. At the age of 38 days (two weeks after weaning) feces were collected of all 32 piglets. For 16 of the 32 animals (8 per feeding group) an additional digesta and mucosa sample per piglet were collected after the animals were sacrificed. At the age of 52 days (four weeks after weaning) feces of the remaining 16 animals were collected and after the animals were sacrificed, again one digesta and one mucosa sample per animal were collected. The sampling time points could vary by ±2 days due to time consuming sample processing, limited “human resources”, and the fact that before taking gut samples the animals had to be euthanized and several other samples have also been taken by other experimental groups.

Feces were collected from the ampulla recti and digesta and mucosa scrapings were taken from the colon ascendens as described previously [31]. To collect mucosa scrapings, digesta of a 2-5cm long piece intestine was removed by gently massage, intestine was opened by longitudinal incision and after two or three washing steps in 1x phosphate buffered saline (PBS) solution, mucosa was scraped with a sterile slide. Samples from colon ascendens were gathered after sedation [20 mg/kg bodyweight (BW) of ketamine hydrochloride (Ursotamin^®^, Serumwerk Bernburg AG, Germany) and 2 mg/kg BW of azaperone (Stresnil^®^, Jansen-Cilag, Neuss, Germany)] and euthanasia with intracardial injection of 10 mg/kg BW of tetracaine hydrochloride, mebezonium iodide, and embutramide (T61^®^, Intervet, Unterschleißheim, Germany) followed by a midline abdominal incision and removal of the entire intestinal tract from the peritoneum.

### Isolation of *E*. *coli*

The protocol for *E*. *coli* isolation was adapted from Bednorz et al. [20, 31]. Samples were suspended in PBS (mucosa scrapings were homogenized with a dounce homogenizer), serially diluted and plated on sheep blood agar plates (Oxoid, Wesel, Germany), CHROMagar Orientation plates (CHROMagar, Paris, France) as well as CHROMagar Orientation plates supplemented with one of nine antimicrobial substances. Thus, a total of ten CHROMagar Orientation plates with different ingredients were utilized. This extensive initial isolation procedure was chosen to increase the diversity of *E*. *coli* isolates utilized for our in depth analysis. Concentrations of antibiotics were adapted from Guenther et al. [32] or are derived from the breakpoint concentrations of the Clinical and Laboratory Standards Institute [33, 34] (ampicillin 32 μg/ml, streptomycin 64 μg/ml, nalidixic acid 32 μg/ml, sulfamethoxazole-trimethoprim 76 μg/ml / 4 μg/ml, tetracycline 16 μg/ml, cefotaxime 4 μg/ml, gentamicin 16 μg/ml, enrofloxacin 2 μg/ml, or chloramphenicol 32 μg/ml).

After 18–24 hours of aerobic incubation at 37°C, per specimen 7 to 22 colonies with typical dark pink to reddish morphology were randomly chosen from different agar plates (each colony representing a single isolate). Isolates were sub-cultured twice on CHROMagar Orientation and sheep blood agar plates, grown in Lysogeny broth (LB) media (Luria/Miller) (Carl Roth, Karlsruhe, Germany) and stored in 20% glycerol (Carl Roth, Karlsruhe, Germany) stocks at -80°C for further investigation.

### Quantification of *E*. *coli*

Colonies with typical dark pink to reddish morphology were counted for each of the ten different CHROMagar Orientation plates (one without supplementation and nine with antimicrobial substances added). The colony forming units (cfu)/g of each sample were compared for the different habitats, time points, and agar plates between the two feeding groups. In addition the cfu for each of the three habitats were compared in regard to the different sampling time points. For the cfu/g feces the time points 24 days of age (before weaning) and 52 days of age were compared for the 16 animals which were sacrificed after 52 days of age and for digesta and mucosa cfu values were compared between 38 days of age and 52 days of age for the sacrificed animals.

### Screening for phenotypic resistance and multi-resistance

**Fig 2:**
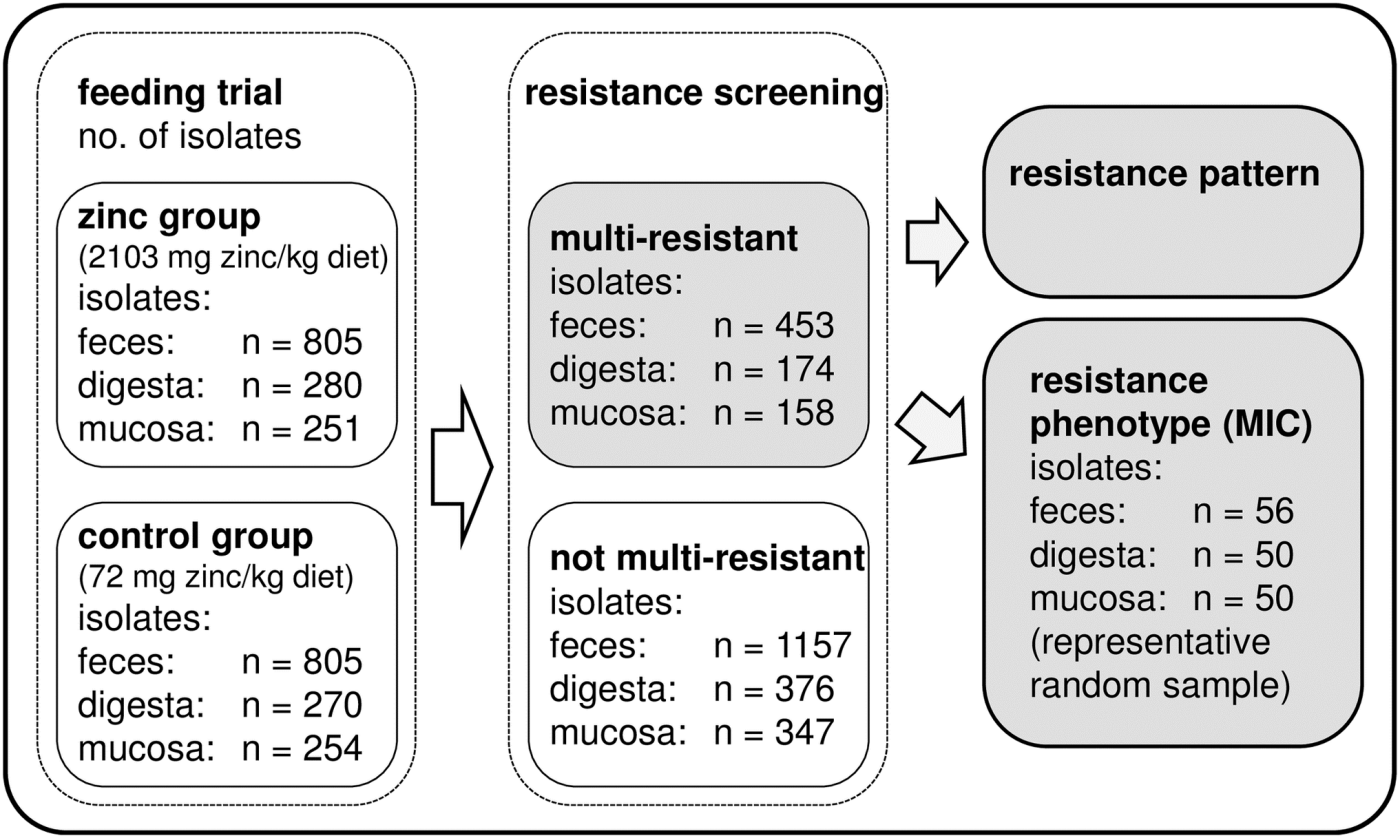
Schematic workflow of the analyses. Number of isolates investigated in this study in association to the multi-resistance status, the different investigations, and the three different habitats. MIC: minimum inhibitory concentration.

Our extensive sampling strategy amounted to a total number of 2.665 *E*. *coli* isolates (1610 from feces, 550 from digesta, and 505 from mucosa, respectively Fig 2). To analyze these isolates, we adopted a protocol for phenotypic resistance screening of large numbers of isolates [32]. After overnight culture on sheep blood agar plates isolates were sub-cultured on LB agar plates supplemented with one antimicrobial substance (ampicillin, streptomycin, sulfamethoxazole-trimethoprim, tetracycline, enrofloxacin (concentrations as mentioned above), or kanamycin 64 μg/ml). Agar plates were incubated overnight at 37°C and growth was evaluated visually. Isolates were defined as multi-resistant when they were resistant against at least antimicrobial substances of three different antimicrobial classes [35].

### Validation of the screening by MIC determination

To validate the screening method, we analyzed a representative random sample by minimum inhibitory concentration (MIC) determination for ampicillin, sulfamethoxazole-trimethoprim, tetracycline, and enrofloxacin using Micronaut-S livestock plates according to the supplier’s protocol (Merlin, Bornheim-Hersel, Germany). This representative random sample of multi-resistant isolates for each habitat was created using http://epitools.ausvet.com.au/. Calculation of sample sizes was based on a representative freedom survey with the parameters prevalence 5% and population sensitivity 95%. Consequently 56 feces, 50 digesta, and 50 mucosa isolates were investigated.

### Statistics

Quantitative data for *E*. *coli* (cfu/g sample) were compared between the two feeding groups and for digesta and mucosa between the two time points 38 and 52 days of age based on results of the Mann-Whitney U test [36] as two independent groups for which normal distribution could be rejected were investigated. The cfu/g feces between the time points 24 and 52 days of age were compared by paired-samples sign test [37] as again normal distribution could be rejected but this time we investigated paired observations with two measured values for the same 16 piglets. The proportion of resistant and multi-resistant *E*. *coli* between feeding groups and over time was compared with the Pearson chi-square-test [38] as binary data of independent groups were investigated. Investigation of normal distribution was performed with the Kolmogorov-Smirnov test. We did not correct for multiple testing as each performed statistical test checked a different hypothesis. Different habitats as well as different time points were therefore investigated separately. Statistical analyses and randomized sampling of the isolates for MIC determination were performed with IBM SPSS Statistics version 20.

## Results

### Quantitative analysis of the *E*. *coli* population

**Fig 3:**
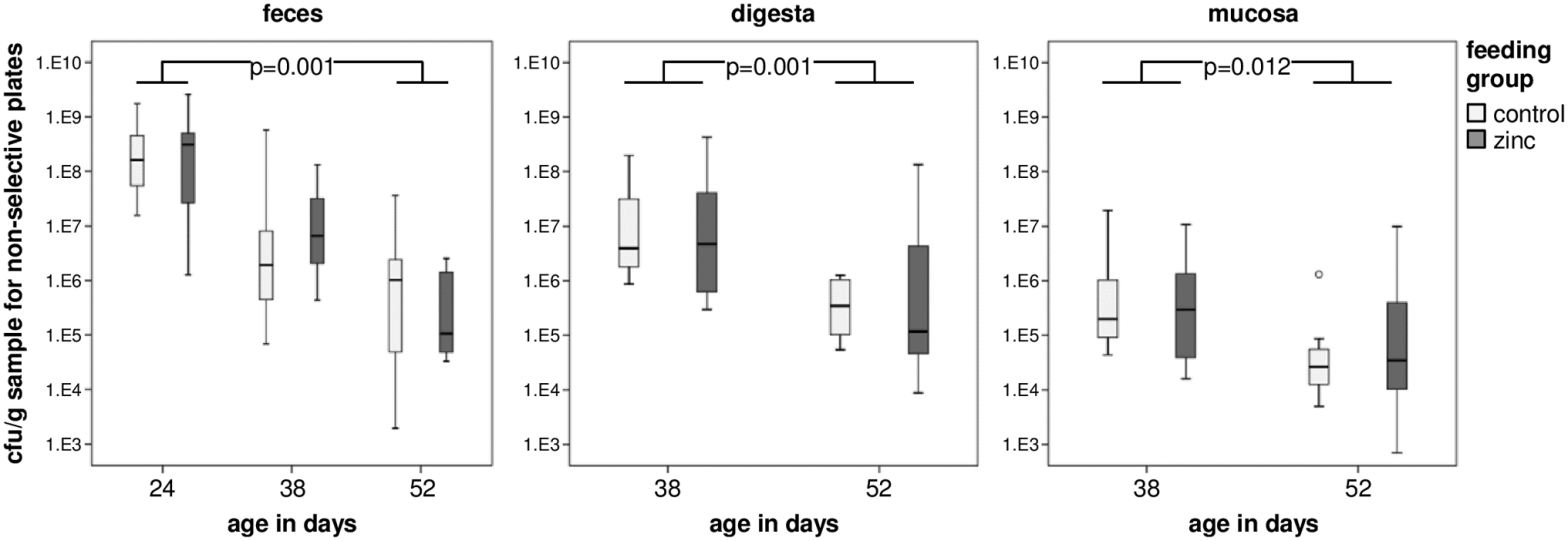
Absolute numbers of the porcine *E*. *coli* population. Quantitative data for the *E*. *coli* isolates of the zinc feeding trial in association to intestinal habitat (feces, digesta, mucosa) and age of piglets (24, 38, 52 days of age). Colonies were counted on CHROMagar Orientation plates and the cfu/g sample was determined. Significant differences are indicated by p values and could only be detected for changes between different time points but not between the two feeding groups. Outliers are displayed separately.

Initially, the absolute numbers of *E*. *coli* were determined for each of the three different time points (24, 38, and 52 days of age) as well as each of the three different habitats (feces, digesta, and mucosa). The cfu/g sample varied from approximately 10^4^ to 10^9^ (feces), 10^4^ to 10^8^ (digesta), and 10^3^ to 10^7^ (mucosa), respectively (Fig 3, S1 Table), regardless from which feeding group the *E*. *coli* had been isolated from. In addition we observed a substantial decrease of the *E*. *coli* population after weaning in all three habitats (feces paired-samples sign test; p = 0.001, digesta Mann-Whitney U test; p = 0.001, mucosa Mann-Whitney U test; p = 0.012). In summary, no significant differences between the control and the zinc group in the absolute number of *E*. *coli* grown on non-selective agar plates was detectable, neither in regard to the sampling time points or the habitats.

Regarding *E*. *coli* colonies grown on selective agar plates supplemented with antimicrobial substances, again we observed no significant difference in the number of *E*. *coli* between the control and zinc group across different time points, habitats, or the nine different antimicrobial supplements (data not shown).

### Isolation of *E*. *coli* and screening for antimicrobial resistance

Up to 22 *E*. *coli* colonies per sample were isolated from different agar plates resulting in a total of 1610 feces, 550 digesta, and 505 mucosa isolates, distributed equally between the two feeding groups. Isolates were screened for their phenotypic resistance against six antimicrobial substances of five different antimicrobial classes (S2 Table).

For isolates resistant against at least one antimicrobial substance significant differences between the feeding groups could only be detected for feces and digesta isolates. In detail, 66.8% of feces isolates from the zinc group were resistant and only 60.4% of the control group. With respect to the three different time points only at the age of 38 days a significant difference was detectable between the two feeding groups with 69% resistant isolates in the zinc group and 57.6% in the control group (chi-square-test; p = 0.004). For digesta 57.1% isolates of the zinc group and 44.1% of the control group were resistant (chi-square-test; p = 0.002). This time a significant difference was detectable at the age of 38 days (78% resistant isolates in zinc group, 63.5% in control group, chi-square-test; p = 0.007) and at the age of 52 days (36% resistant isolates in zinc group, 21.6% in control group, chi-square-test; p = 0.01). For mucosa 53.4% of isolates from the zinc group and 47.6% of the control group were resistant. In general the proportion of isolates which were resistant against at least one antimicrobial substance decreased in both feeding groups and for all different habitats over time (feces, digesta, mucosa: chi-square-test, p<0.001).

**Fig 4:**
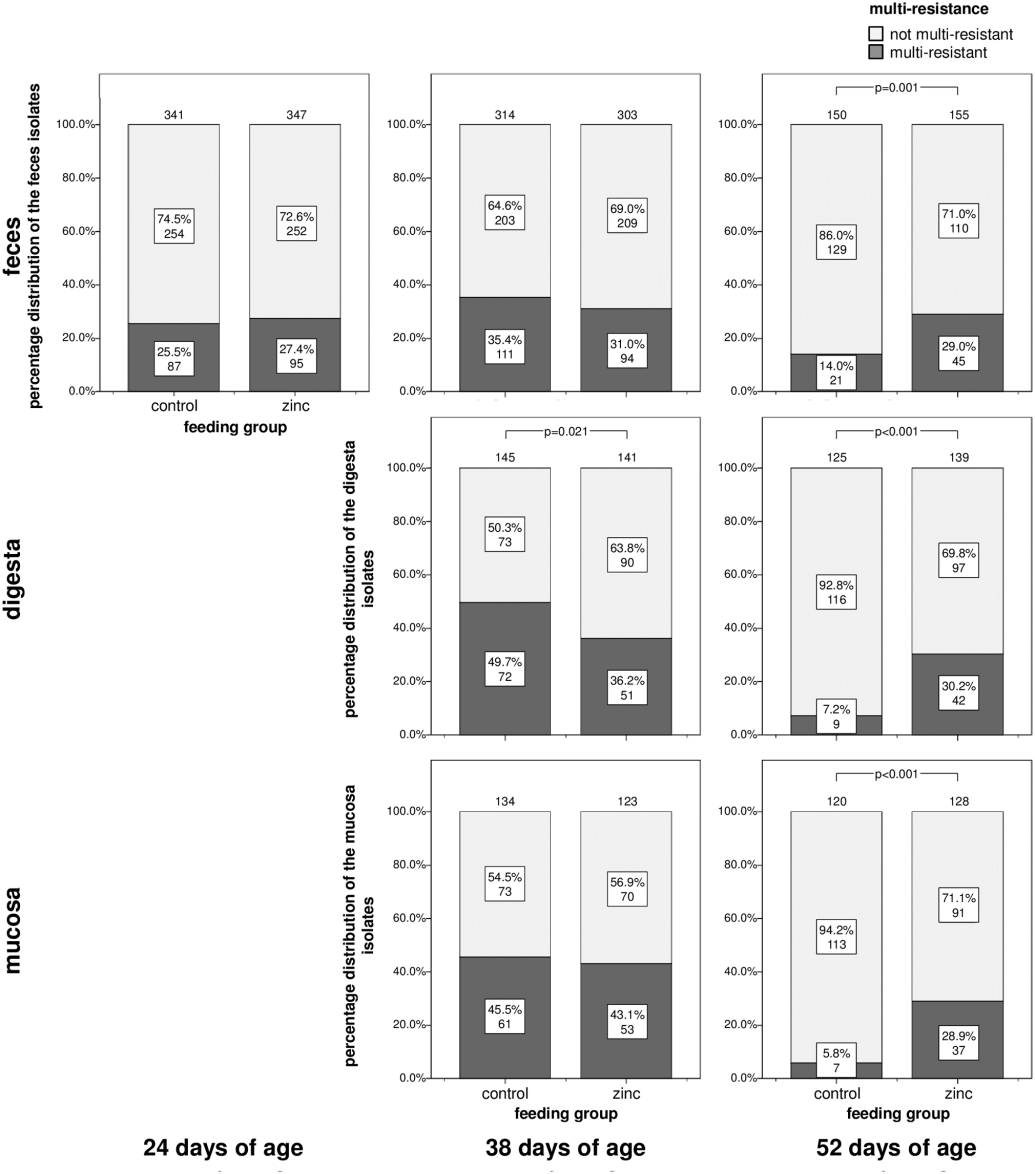
Proportion of multi-resistant porcine *E*. *coli* isolates. Piglets were weaned at 25±1 days of age and data for the multi-resistance status are shown for feces, digesta, and mucosa isolates of the three sampling time points (24, 38, 52 days of age) and for both zinc feeding groups of the trial. Phenotypic resistance against six different antimicrobial substances was determined and isolates were assigned as multi-resistant when they were resistant against at least three different antimicrobial classes. Numbers indicate the number of investigated isolates. Significant differences are indicated by p values.

In contrast, analyses of multi-resistant isolates revealed a strong association to high dietary zinc feeding in all three tested habitats at the age of 52 days with a higher proportion of multi-resistant isolates in the zinc group (Fig 4). In detail for feces isolates, directly before weaning (24 days of age) and at the age of 38 days the proportion of multi-resistant isolates did not differ between the two feeding groups (chi-square-test; 24 days of age p = 0.579, 38 days of age p = 0.254) but at the age of 52 days we observed a highly significant difference with 29% multi-resistant isolates in the zinc group and only 14% multi-resistant isolates in the control group (chi-square-test; p = 0.001).

The same was true for digesta isolates from the same age where 30.2% multi-resistant isolates occurred in the zinc group, but only 7.2% in the control group (chi-square-test; p<0.001). Interestingly, a significant difference was also detectable at the age of 38 days, whereas this time the proportion of multi-resistant isolates was higher in the control group (36.2% multi-resistant isolates in the zinc group and 49.7% in the control group, chi-square-test; p = 0.021).

Finally for mucosa isolates comparable results were observed with no significant differences at the age of 38 days (chi-square-test; p = 0.695) and a highly significant difference at the age of 52 days (28.9% multi-resistant isolates in the zinc group and 5.8% in the control group, chi-square-test; p<0.001).

Overall the proportion of multi-resistant isolates in the control group decreased in all habitats after 38 days of age whereas in the zinc group, multi-resistant isolates maintained at a higher proportion.

### Validation of the screening by MIC determination

As outlined in Materials and Methods, due to the high number of isolates we determined MIC values of multi-resistant isolates against ampicillin, sulfamethoxazole-trimethoprim, tetracycline, and enrofloxacin by stratified random samples (prevalence 5% and population sensitivity 95%). This resulted in an investigation of 56 out of 453 feces, 50/174 digesta, and 50/158 mucosa multi-resistant isolates. Overall MIC determination confirmed the results of the phenotypic screening as MIC values were higher as the breakpoints which were used for the resistance screening. For all tested isolates the results of the screening were confirmed, with the exception of two mucosa isolates. One isolate which was resistant against sulfamethoxazole-trimethoprim 76 μg/ml / 4 μg/ml in the screening assay had a MIC value for sulfamethoxazole-trimethoprim of ≤4.75 μg/ml / 0.25 μg/ml. The second isolate was resistant against tetracycline 16 μg/ml in the screening assay but had only a MIC value of tetracycline ≤1 μg/ml.

### Resistance pattern

To stratify between different multi-resistant populations, the 453 feces, 174 digesta, and 158 mucosa multi-resistant isolates were grouped according to their resistance patterns. We therefore analyzed the percentage distribution with regard to the different habitats and sampling time points.

**Fig 5:**
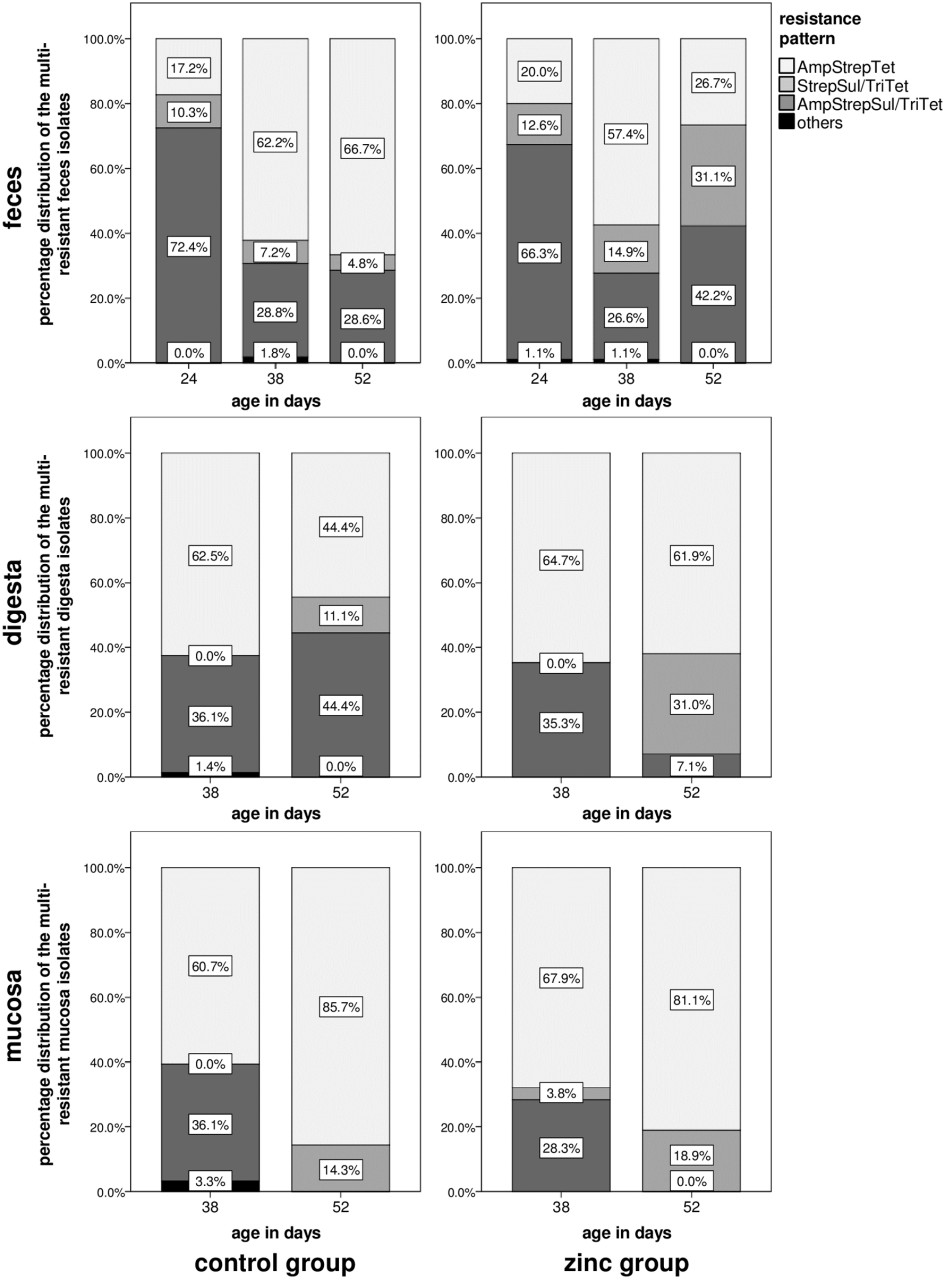
Distribution of the phenotypic resistance patterns of the 453 feces, 174 digesta, and 158 mucosa multi-resistant *E*. *coli* isolates. Multi- resistance was defined as resistance against at last three classes of antimicrobials. The distribution was analyzed for the different habitats (feces, digesta, mucosa) and sampling time points (24, 38, 52 days of age) for both feeding groups of the zinc pig feeding trial.

As outlined in Fig 5, in all three habitats three main resistance patterns were observed, differing in their proportions in each habitat and over time.

For isolates from the habitat feces at the age of 24 days, the three main different resistance patterns namely resistance against 1. ampicillin, streptomycin, sulfamethoxazole-trimethoprim, and tetracycline (AmpStrepSul/TriTet), 2. streptomycin, sulfamethoxazole-trimethoprim, and tetracycline (StrepSul/TriTet), or 3. ampicillin, streptomycin, and tetracycline (AmpStrepTet) did not differ in their proportion between the zinc and the control group. At the age of 38 days the resistance patterns were again nearly identical between the two groups with the exception that resistance pattern StrepSul/TriTet increased in the zinc group compared to the control group. But substantial changes in the resistance pattern were detectable at the age of 52 days. Whereas in the control group the resistance pattern did not change, in the zinc group the resistance patterns AmpStrepSul/TriTet and StrepSul/TriTet gained a higher proportion and pattern AmpStrepTet decreased. Especially pattern StrepSul/TriTet had a dramatic change with 4.8% proportion in the control group and 31.1% in the zinc group.

For digesta isolates we observed a partly comparable change in the distribution pattern. At the age of 52 days a clear difference between the two feeding groups was detectable. Like the feces isolates the proportion of pattern StrepSul/TriTet was increased in the zinc group (31% of the isolates) compared to the control group (11.1% of the isolates). However, this time pattern AmpStrepSul/TriTet decreased in the zinc group (7.1% of the isolates vs. 44.4% of the isolates in the control group). For mucosa the distribution of the different resistance patterns looked very similar for both feeding groups with pattern AmpStrepTet as the dominant pattern. At the age of 38 days resistance pattern AmpStrepSul/TriTet was the second dominant pattern whereas at the age of 52 days only resistance pattern StrepSul/TriTet coexisted beside pattern AmpStrepTet.

## Discussion

We have recently been able to show that feeding high zinc concentrations to weaned piglets strongly increases the proportion of multi-resistant *E*. *coli* [20]. In the previous study none of the *E*. *coli* clones isolated from the control group but 18.6% of those clones isolated from the zinc group were multi-resistant. This finding could have a massive influence on the current feeding practices of zinc for weaned piglets as it scrutinizes the meaningfulness of high dietary zinc feeding as a substitute for antimicrobial growth promoters.

Therefore we aimed to falsify our previous results in an independent second study, using the same feeding setup, but changing the experimental setup focusing on a complex analysis on resistance phenotypes rather than clonal diversity, as we had done previously. Regardless of the changed setup, again, we observed that high dietary zinc feeding increases the proportion of multi-resistant *E*. *coli* isolates of weaned piglets, corroborating the finding of the previous study. However, the modified trial set up allowed deeper insights into the population dynamics of the pigs gut. Here, we were able to show that the proportional increase of multi-resistant *E*. *coli* is an effect of persistence of resistant *E*. *coli* populations over time rather than an increase of multi-resistant isolates. Notably, this effect was independent from the total numbers of *E*. *coli*, meaning that the total amount of the intestinal *E*. *coli* population was not affected by zinc feeding. In addition we observed this zinc effect in all three habitats tested (feces, digesta, and mucosa), strongly substantiating a major effect of high dietary zinc feeding, as it is known that a single habitat does not fully reflect the microbiota of different intestinal niches [31].

The proportion of multi-resistant isolates remained stable for both feeding groups before weaning until the age of 38 days but we detected dramatic changes at the age of 52 days. In contrast to a decreasing proportion of multi-resistant isolates in all control groups, we observed stable numbers of multi-resistant isolates in the zinc groups of all habitats. As the overall *E*. *coli* population decreased in both feeding groups, high dietary zinc feeding seems to increases the persistence of multi-resistant strains.

What are possible explanations for these findings? Can multi-resistant *E*. *coli* better adapt to the stress of weaning and the following changes in the microbiota of the gut under the influence of high dietary zinc? This would be rather surprising, as multi-resistance is often conferred via large resistance plasmids which have been linked to a reduction of bacterial fitness in non-selective surroundings [39, 40]. However there is growing evidence that fitness loss is not the case at least for certain successful bacterial lineages [41–43], probably due to compensatory mutations [44]. Furthermore, if multi-resistance would lead to a general fitness disadvantage the observed decrease of the overall *E*. *coli* population over time should have caused an early decrease of the multi-resistant population as well. Interestingly this effect was only observed for the control group. This particular effect of high dietary zinc feeding on multi-resistance was only detectable at the piglets’ age of 52 days and might be due to the well-known nutritional changes after weaning. As weaning has a massive influence on the microbiome [45, 46] we speculate that this effect could only be detected after the microbiome stabilized over time. We have to state that a feeding duration of four weeks is a long period, and it can be argued that this long feeding time is a necessity to cause the observed changes. We were not able to determine an effect of zinc feeding on multi-resistance two weeks after weaning (at 38 days of age). However, weaning associated changes in the microbiome are detectable longer than two weeks after weaning [47, 48]. Therefore we speculate that also shorter feeding durations lead to an increased proportion of multi-resistant *E*. *coli* which is only detectable after the microbiome has stabilized over time. Regardless of these aspects, in practice zinc is often fed for longer time periods than 14 days, especially in pig herds with high pathogen pressure and recurrent periods of diarrhea. In the light of the consistent picture with higher proportions of multi-resistant isolates in the zinc group at the age of 52 days, the finding that for digesta isolates at the age of 38 days a higher proportion of multi-resistant isolates was detected in the control group raises questions. It looks like under the weaning associated changes in the microbiome multi-resistant isolates had an advantage in the digesta of the control group. However, neither these populations were able to persist in the digesta over time nor significant differences of the proportion of multi-resistant isolates were detectable between both feeding groups for the two other habitats at the age of 38 days.

In general we observed a higher proportion of multi-resistant isolates compared to our recent work [20]. Presumably this is due to focusing in the current study on resistant isolates, as well as the inclusion of streptomycin into the screening and the fact that streptomycin resistance has become very abundant in *E*. *coli*. These differences in the trial setup may also be the explanation for the divergences in the analysis of isolates resistant against one or more substances between both trials. Whereas this study detected an increasing effect of high dietary zinc feeding for digesta isolates, our previous study identified only slight changes between the feeding groups for digesta clones [20].

In parallel to the resistance status of the *E*. *coli* isolates we investigated the absolute *E*. *coli* numbers. This was of interest as higher bacterial counts would theoretically cause closer contacts between bacterial cells, thus enabling an increased conjugational transfer of plasmids between strains [49]. If these plasmids harbor resistance genes, this obviously would also lead to an increased detection rate of resistant *E*. *coli*. As we did not detect significant differences in the total numbers between the control and the zinc group (in all three habitats as well as at all three sampling time points), our results indicate that the increased proportion of multi-resistant isolates in the zinc group was not an effect of an overall increased *E*. *coli* abundance.

Recently 16SrRNA gene quantification gave evidence that high dietary zinc feeding increases the relative abundance of *E*. *coli* in the digesta microbiota of the porcine ileum [50]. This effect might be limited to the ileum since differences between different intestinal sections have been reported. In addition 16SrRNA gene quantification does not allow to distinguish between viable and dead bacteria. Whatever the reasons for these findings are, they do not contradict our findings.

Interestingly we observed a strong decrease of the overall *E*. *coli* population in both feeding groups in all three habitats at the time closely after weaning. During the post-weaning period changes in Enterobacteriaceae counts depend on factors such as environment, weaning age, and diet [46]. Different studies observed time dependent increasing as well as decreasing, or stable *E*. *coli* populations after weaning [51, 52]. However weaning surely results in stress for the bacterial population in the gut. Our data suggest that the additional influence of high dietary zinc feeding seems to modulate the success of different *E*. *coli* populations during this process.

The analysis of resistance patterns of multi-resistant isolates proves that different intestinal niches display different results. Although the changes in the proportion of multi-resistant isolates were comparable, the resistance patterns showed different results for all three habitats indicating differences in the *E*. *coli* populations of these habitats.

Changes in the resistance pattern could be mainly explained by two effects: (i) one population with a specific resistance pattern has an advantage and is able to outcompete others or (ii) the exchange of mobile genetic elements encoding for these patterns is altered. We clearly need to address these questions by future whole genome sequence analysis to get further insights into the underlying mechanism.

Although a possible connection between heavy metals, heavy metal resistance, and antimicrobial resistance has been discussed widely [22], the mechanisms remain unclear.

Among the possible mechanisms which select for resistant bacteria under the influence of zinc are different mechanisms of co-selection. For Gram-positives it has been shown that in the presence of zinc the genetic co-localization of the *czrC* gene conferring zinc resistance and the *mecA* gene which confers resistance to beta-lactam antimicrobials on the *Staphylococcus* Cassette Chromosome *mec* (SCC*mec*) [53, 54] may select for methicillin-resistant *Staphylococcus aureus* (co-resistance) [55]. However, in our recent study from Bednorz et al. we did not observe a general association of zinc tolerance and antimicrobial resistance [20].

Another mechanism is co-regulation. For *E*. *coli* strain MG1655 an upregulation of the *mdtABC* operon under the influence of excess zinc was shown [56]. This resistance-nodulation-cell division (RND-type) efflux system has been implicated in conferring resistance to certain antimicrobial substances such as novobiocin and the bile salt component deoxycholate [57]. An additional co-regulation mechanism was found for *Pseudomonas aeruginosa* were the presence of zinc activates the expression of the CzcRS two component system, which activates the expression of the metal efflux pump *czcCBA* and suppresses the expression of the OprD porin, the entry porin of carbapenem antibiotics [58]. However, co-regulation is unlikely to explain the observed changes as (i) piglets were raised without exposure to antimicrobial substances and (ii) the phenotypic resistance screening of the isolates was conducted in the absence of zinc.

As antimicrobial resistance is often conferred via transferable plasmids other possible mechanism involved could be (i) an influence of zinc on the plasmid uptake rate by conjugation [59, 60] or (ii) an alteration of bacterial cell membranes by increased expression of genes involved in membrane structure and transport [56]. Indeed we observed differences in the plasmid profiles of clones of the zinc and control group which could be connected with differences in the resistance profile during our recent study [20]. In addition the strong effect of high dietary zinc feeding on especially multi-resistant isolates corroborates the idea of an involvement of plasmids, as these often carry multiple resistance genes conferring resistance to different classes of antimicrobials.

Besides these putative mechanisms, all directly targeting the *E*. *coli* population, it is necessary to keep in mind the complex composition of the intestinal microbiota. It might be that high dietary zinc feeding does not influence e.g. the direct conjugation rate but rather has an influence on intestinal populations other than *E*. *coli*. This could result in new niches for resistant *E*. *coli* populations which adapt faster to changes in the intestinal microbiota. Also, the initial microbiota composition before weaning might play an essential role for the observed changes. As the sows of this trial originate from the same farm as the sows from our recent trial it is also necessary to further investigate the influence of high dietary zinc feeding on resistance with animals of different origins. And finally it might be that zinc effects on the piglets as hosts lead to the detected persistence of multi-resistant *E*. *coli*. Different studies identified several intestinal as well as extra intestinal effects of high dietary zinc feeding on piglets, for example on intestinal morphology, intestinal mucin types, intestinal immune system, hepatic proteome, and methylation [61–64].

Besides the association of high dietary zinc feeding with antimicrobial resistance found by us and others the general data on growth performance of weaning piglets are contrary and therefore the usage of high levels of zinc oxide as feed supplement is highly questionable. Several studies detect a positive influence of therapeutic dosages of zinc oxide on growth performance [65–68]. However, it needs to be mentioned that other studies did not find such a positive influence of high dietary zinc oxide [27, 69, 70], presumably due to different experimental setups.

Taken together the results of this study fully corroborate our previous results where we detected a massive increase in multi-resistant *E*. *coli* isolates under the influence of high dietary zinc feeding after weaning. The same results could be obtained by focusing the sampling schedule on antimicrobial resistant *E*. *coli* thereby ruling out any influence of the clonal classification during the first study. Secondly the increase of multi-resistant *E*. *coli* isolates under the influence of high dietary zinc feeding is not caused by an overall increase of the *E*. *coli* population in the zinc group. In contrary we observed an overall decrease of the *E*. *coli* population after weaning in both groups. In addition we were also able to demonstrate the increasing effect of high dietary zinc feeding on multi-resistant *E*. *coli* for two additional gut habitats.

In conclusion, we suggest that high dietary zinc feeding of weaned piglets promotes the abundance of multi-resistant bacteria. Summing up the data currently available indicate that the usage of high dietary zinc as an alternative for antimicrobial growth promoters is highly questionable and thus inappropriate. Besides the intestinal resistance effect, the positive influence of high dietary zinc feeding for weaning piglets on growth performance seems to be inconsistent and to be dependent on to many variables and third, resistant bacteria, antimicrobial resistance genes, and high concentrations of zinc could be transferred via pig manure on agricultural fields and thus to the environment [71].

In addition, the finding that the feeding of high dietary zinc interferes with antimicrobial resistance highlights the necessity to use holistic approaches in antimicrobial resistance research to control this world wide emerging problem. Besides the urgent need of reducing the usage of antimicrobial substances, the results of our study clearly indicate the existence of additional factors which contribute to the spread of resistant bacteria. Apart from the focus on heavy metals like zinc, future research should also include work on the influence of tenacity to disinfectants or virulence factors and metabolic features to mention a few to combat the spread of antimicrobial resistance.

## Supporting information

S1 Table Quantitative *E*. *coli* data of the zinc pig feeding trial. (XLSX)

S2 Table Data of the antimicrobial resistance screening of the zinc pig feeding trial. (XLSX)
